# Immunoadjuvant therapy in the regulation of cell death in sepsis: recent advances and future directions

**DOI:** 10.3389/fimmu.2024.1493214

**Published:** 2024-12-10

**Authors:** Md. Monirul Islam, Eizo Watanabe, Umme Salma, Masayuki Ozaki, Takayuki Irahara, Subaru Tanabe, Ryusuke Katsuki, Dai Oishi, Naoshi Takeyama

**Affiliations:** ^1^ Department of Emergency and Critical Care Medicine, Aichi Medical University, Nagakute, Japan; ^2^ Department of Biochemistry and Biotechnology, University of Science and Technology Chittagong (USTC), Chattogram, Bangladesh

**Keywords:** apoptosis, autophagy, NETosis, pyroptosis, ferroptosis, necroptosis cell death, immunosuppression, sepsis

## Abstract

Sepsis is characterized by a concomitant early pro-inflammatory response by immune cells to an infection, and an opposing anti-inflammatory response that results in protracted immunosuppression. The primary pathological event in sepsis is widespread programmed cell death, or cellular self-sacrifice, of innate and adaptive immune cells, leading to profound immunological suppression. This severe immune dysfunction hampers effective primary pathogen clearance, thereby increasing the risk of secondary opportunistic infections, latent viral reactivation, multiple organ dysfunction, and elevated mortality. The types of cell death include apoptosis (type I programmed cell death), autophagy (type II programmed cell death), NETosis (a program for formation of neutrophil extracellular traps (NETs)) and other programmed cell deaths like pyroptosis, ferroptosis, necroptosis, each contributing to immunosuppression in distinct ways during the later phases of sepsis. Extensive apoptosis of lymphocytes, such as CD4+, CD8+ T cells, and B cells, is strongly associated with immunosuppression. Apoptosis of dendritic cells further compromises T and B cell survival and can induce T cell anergy or promote regulatory Treg cell proliferation. Moreover, delayed apoptosis and impaired neutrophil function contribute to nosocomial infections and immune dysfunction in sepsis. Interestingly, aberrant NETosis and the subsequent depletion of mature neutrophils also trigger immunosuppression, and neutrophil pyroptosis can positively regulate NETosis. The interaction between programmed cell death 1 (PD-1) or programmed cell death 1 ligand (PD-L1) plays a key role in T cell modulation and neutrophil apoptosis in sepsis. The dendritic cell growth factor, Fms-like tyrosine kinase (FLTEL), increases DC numbers, enhances CD 28 expression, attenuates PD-L1, and improves survival in sepsis. Recently, immunoadjuvant therapies have attracted attention for their potential to restore host physiological immunity and homeostasis in patients with sepsis. This review focuses on several potential immunotherapeutic agents designed to bolster suppressed innate and adaptive immune responses in the management of sepsis.

## Introduction

Sepsis is characterized by a dysregulated inflammatory host response to life-threatening infection, which can trigger circulatory shock, organ dysfunction, and ultimately, death (1). The hallmark of sepsis is a simultaneous, cytokine-mediated, early excessive pro-inflammatory response to infection that contributes to the recruitment and activation of innate and adaptive immune cells to the site of infection. Progression of sepsis leads to immune cell exhaustion by continuous encounters with pathogens and inflammatory signals with increased expression of immune checkpoint molecules like PD-1 and CTLA-4. The exhausted immune cells exert a compensatory anti-inflammatory response and undergo different types of massive cell death (2). The human body manages this through a negative feedback mechanism referred to as immunosuppression, which leads to immunoparalysis involving both the innate and adaptive immune systems. This profound immune dysfunction results in poor primary pathogen clearance and enhances the risk of secondary opportunistic infections, latent viral reactivation, multiple organ dysfunction, and increased mortality (3, 4). Despite numerous clinical trials focused on mitigating hyper-inflammation by blocking pro-inflammatory mediators (5, 6), no FDA-approved treatments have been approved to date, and sepsis remains a predominant cause of death among critically ill patients in most intensive care settings worldwide (7). While advances in treatment and supportive care have reduced mortality and improved overall survival in sepsis management, researchers have yet to elucidate various immunological aspects of the syndrome or identify novel, targeted therapeutics to reverse sepsis effectively. It is well established that cell death, a conserved mechanism in multicellular organisms, plays a vital role in responding to external injuries. Dysregulation of widespread programmed immune cell death, or cellular self-sacrifice, is now recognized as the primary pathological event in sepsis, leading to significant immunological suppression (8). These cell death mechanisms include apoptosis (type I programmed cell death), autophagy (type II programmed cell death), NETosis [a program for formation of neutrophil extracellular traps (NETs)] pyroptosis, ferroptosis, and necroptosis. An inadequate immune response resulting from cell death and subsequent aggressive immunosuppression has been identified as a significant contributor to sepsis pathogenesis (9, 10). Excessive apoptosis of splenic CD4^+^, CD8^+^ T, and B cells, coupled with reduced autophagy in CD4^+^ T cells, has been observed in patients with sepsis, thereby accelerating acquired immunodeficiency (11). Furthermore, apoptosis of dendritic cells in sepsis also compromises the survival of T cell and B cells and can induce a state of T cell anergy or promote regulatory T cell (Treg) proliferation (12). In addition, during sepsis, the major interferon-gamma (IFN-γ)- producing natural killer (NK) cells encounter immoderate apoptosis after a reduced number is present in the circulation, thus increasing the risk of secondary infection (13). Due to the inhibition of spontaneous apoptosis, neutrophils may undergo other types of cell death, including NETosis and pyroptosis (14). Excessive NETosis, followed by the depletion of mature neutrophils, heightens the risk of nosocomial infections and immune dysfunction. Investigators are actively trying to clarify the underlying inconsistencies in innate and adaptive immunity as well as the mechanisms of immunosuppression that contribute to the long-term prognoses in sepsis. Consequently, immunoadjuvant therapies have recently attracted considerable attention in sepsis management to restore host immunity. This review focuses on the dysregulation of immune cell death patterns induced by sepsis and the subsequent disruptions to immunity. In addition, we outline current potential therapeutic interventions, including interleukin (IL)-7, IL-15, IFN-γ, granulocyte-macrophage colony stimulating factor (GM-CSF), Fms-like tyrosine kinase-3 ligand, inhibition of programmed cell death protein 1(PD-1), programmed cell death ligand 1 (PD-L1), and other cell death checkpoints, as well as future directions for sepsis management.

## Mechanism of immune cell deaths in sepsis

The prime mechanism of immune cell death in sepsis is a type I programmed cell death, apoptosis. To date, three pathways of apoptosis have been reported: the extrinsic (death receptor) pathway, the intrinsic (mitochondrial) pathway, and the perforin/granzyme pathway. The three pathways intersect into the common execution pathway initiated by activating the effector enzyme cysteinyl aspartate- specific protease (caspase)-3 (15). Tumor necrosis factor (TNF)-α, high mobility group box-1 protein (HMGB1), Fas ligand (FasL), heat shock, oxygen-free radicals, nitric oxide (NO), glucocorticoids, granzymes, and TNF-alpha-induced protein eight like-2 (TIPE2) are the triggers of apoptosis, on the contrary interleukin (IL)-1, IL-6, and granulocyte colony-stimulating factor (G-CSF) are the inhibitor of apoptosis (16). The extrinsic pathway of apoptosis involves interaction between TNF family-derived extracellular death ligands, e.g., FasL, TNF-α, and the corresponding death receptors that include Fatty acid synthetase receptor, FasR, TNFR1. The ligand-receptor binding induces the recruitment of cytoplasmic adaptor protein FADD for FasL/FasR and TRADD with the recruitment of FADD and RIP in the case of TNF-α/TNFR1. Currently, caspase-8 gets activated by forming a death-inducing signaling complex (DISC) through the association of FADD and procaspase-8 (17). Caspase-8 activation leads to the downstream execution phase of apoptosis. The intrinsic pathway involves a wide range of non-receptor-mediated stimuli, and the intracellular signal generated changes the inner mitochondrial membrane. This pathway is governed by anti-apoptotic versus pro-apoptotic Bcl-2 family members. Bcl-2, Bcl-x, Bcl-XL, Bcl-XS, Bcl-w, BAG, Mcl-1, and Bfl-1/A1 are anti-apoptotic proteins, and Bcl-10, Bak, Bax, Bim, Bik, Blk, Bmf, Bad, and Bid are pro-apoptotic protein. These proteins determine whether the cell will undergo cell death or skip the death process. The intrinsic and extrinsic pathways are associated, and the molecules involved in each pathway can impact each other.

NETosis is a novel cell death program distinct from apoptosis and necrosis (14). In NETosis stimulated neutrophil release, NETs, a web-like architecture composed of a DNA backbone decorated with anti-microbial proteins like myeloperoxidase (MPO), neutrophil elastase (NE), and cathepsin G (18, 19). Although NETosis is a physiological process and essential part of the innate immune system to eliminate invading microbes, but the uncontrolled NETosis has a pathological role in numerous ways, attributed to sepsis, autoimmune, infectious, and non-infectious diseases that have attracted recent attention. NETosis occurs via two pathways: suicidal NETosis and vital NETosis. Suicidal NETosis is a lytic and slow cell death process, usually taking 2–4 h, whereas vital NETosis is a cell-death-independent non-lytic process that happens faster, within 5–60 min (20). To date, two different mechanisms, NADPH Oxidase 2 (Nox 2)-dependent and Nox 2-independent of NETosis, have been validated. Nox-dependent NETosis is triggered by inducers like PMA, LPS, and bacteria such as *Pseudomonas aeruginosa*, while agonists like calcium ionophores (A231128, ionomycin), uric acid crystals, certain microbes, and UV light trigger Nox-independent NETosis through the formation of different ROS, Nox-ROS and mitochondrial ROS, respectively (21). Different sets of kinases (MAPK, ERK, p38, and JNK) specific to both NETosis get activated, leading to transcriptional firing and activation of downstream pathways. In both types of NETosis ultimately nuclear membrane disintegrates, and NETs are expelled.

Pyroptosis is a caspase-1 (canonical pathway) or caspase-4/5/11(non-canonical pathway)-dependent proinflammatory programmed cell death process (22). The control form of this cell death is a part of innate immunity to actuate phagocytic immune cells and thus control pathogen infection. On the other hand, exaggerated pyroptosis results in a dysregulated host immune response and augments inflammatory injury, leading to organ dysfunction or septic shock. In the canonical pathway, intracellular pattern recognition receptors (PRRs) such as NLRP1B, NLRP3, NLRC4, recognize the stimulus signals of pathogens and activate caspase-1 protein through the association of pro-caspase-1, and adaptor protein ASC. In the non-canonical pathway, bacterial LPS directly bind and activate caspase-11/4/5 (23). At this point, gasdermin D gets activated, and pyroptosis occurs by rapid cell membrane disruption and release of proinflammatory mediators.

Autophagy, a type II programmed cell death, is an essential cellular process and a damaged protein or organelle degradation system necessary for cellular homeostasis. These regulated innate immune defense mechanisms act as cellular defense against oxidative stress and the elimination of pathogenic microorganisms and play a role in antigen presentation (24). Autophagy begins with the formation of a double-membrane vesicle called autophagosomes. Many signaling complexes and pathways participate in the initiation and maturation of the autophagy process.

Ferroptosis is a unique form of iron-dependent programmed cell death distinct from apoptosis, necrosis, and autophagy (25). In this process, lipid peroxides are generated from intracellular ROS and hydrogen peroxide (H_2_O_2_) by the action of iron and oxidize lipid membranes with polyunsaturated fatty acids (PUFAs) (26). At this stage, membrane damage begins followed by cell death. Innate and adaptive immune cells such as macrophages, T, and B cells undergo ferroptosis, reducing numbers and function. This cell death favors bacterial multiplication and dampens the body’s immune function, leading to sepsis (27).

Necroptosis, a novel form of programmed cell death, plays a significant role in the pathophysiology of sepsis. This death process is initiated by activating death receptors like TNF receptor 1 (TNFR1) (28). Then, the receptor-interacting protein kinase 1 (RIPK1) gets activated, which subsequently phosphorylates and activates RIPK3. The RIPK1-RIPK3-mixed kinase domain-like protein (MLKL) complex facilitates cell death by forming membrane pores. *Staphylococcus aureus* is responsible for nosocomial infection and sepsis (29). *Staphylococcus aureus* can also induce necroptosis of macrophages (30) and neutrophils (31) in host cells.

## Role of immune checkpoint in sepsis

Immune checkpoints are specific membrane molecules and the key controllers of the immune system that balance immune homeostasis and limit excessive immune response. Immune checkpoints play a significant role in the pathophysiology of sepsis (32). Leukocytes (neutrophils, monocytes, natural killer cells, and dendritic cells) and lymphocytes (T and B cells) express checkpoint molecule PD-1 on their surface. PD-1 can interact with complementary ligand PD-L1 on the surface of antigen-presenting cells (APCs) such as monocytes, macrophages, and dendritic cells. Cell surface inhibitory immune checkpoint molecules include PD-1, PD-L1, PD-L2, cytotoxic T lymphocyte antigen-4 (CTLA-4), B and T lymphocyte attenuator (BTLA), lymphocyte activation-gene-3 (LAG-3) and T cell membrane protein-3 (TIM-3) and 2B4 (33). This review will focus on the PD-1/PD-L1 axis. During sepsis, both innate and adaptive immune cells become immunocompromised. PD-1/PD-L1 axis is involved in immune cell dysfunction and sepsis-induced immunosuppression (34). A few studies confirmed that increased PD-L1 expression on neutrophils and monocytes is linked to both pro- and anti-inflammatory cytokine levels, decreased phagocytic capacity, delayed apoptosis of neutrophils, and mortality in septic patients (34, 35). A recent study suggests that overexpression of NK cell PD-L1 is associated with increased sepsis severity (36). Increased levels of PD-1 expression in T cells have been reported to be associated with lymphopenia, T cell death, and increased mortality (37).

## Sepsis-induced innate immune cell death

**Figure 1:**
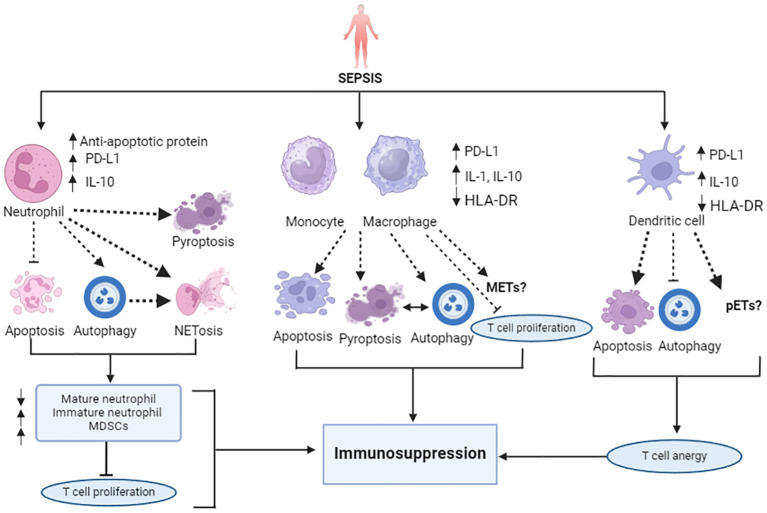
Overview of sepsis-attributed immunosuppression: Impairment of innate immune cell death pathways. In sepsis, alteration of cytokines (upregulated IL-1, IL-10) and reduced antigen presentation (downregulated HLA-DR) is marked. Sepsis slows neutrophil apoptosis and augments NETosis, autophagy, and pyroptosis, like cell deaths, resulting in an increase in the number of immature neutrophils, T cell proliferation inhibitory MDSCs, and the depletion of mature neutrophils. Monocytes and macrophages also encounter increased apoptosis, pyroptosis, autophagy, and NETosis-like cell death. Dendritic cells undergo extensive apoptosis, which induces a tolerogenic state. Unlike increased NETosis, DCs have less potential to undergo autophagy in sepsis. The PD-1/PD L-1 axis plays a significant role in the induction of all these cell death pathways. All these different cell death patterns contribute to immunosuppression in sepsis.

Sepsis markedly affects the lifespan, production, and function of the effector cells within the innate immune system, thereby disrupting homeostasis. The innate immune system, which serves as the body’s front line of defense, consists of neutrophils, monocytes and macrophages, dendritic cells, and other components. Sepsis induces marked losses of these innate immune cells through various cellular death pathways, contributing to immune suppression (**Figure 1**).

Neutrophils, the most abundant circulating leukocytes derived from bone marrow and primary responders to pathogen attack, typically undergo apoptosis within 24 hours of release (38). In sepsis, unlike the delayed apoptosis observed in lymphocytes, mature neutrophils exhibit several dysfunctions: release of immature neutrophils from bone marrow to circulation, reduced oxidative burst capacity, decreased cell migration, diminished complement activation, and impaired bacterial clearance. These factors contribute to the development of immune suppression and persistent inflammation, which may continue even after the disappearance of symptoms (9). This significant impairment of neutrophil functions increases the susceptibility of patients to nosocomial infections (39), ventilator-associated pneumonia (40), and other secondary infections (41). Experiments using a mouse model of sepsis have provided further support for these findings, demonstrating reduced neutrophil functions and increased risk of secondary *Pseudomonas aeruginosa* infection (42) and organ injury (43). Prolonged neutrophil survival is attributed to an imbalance between anti-apoptotic and pro-apoptotic signals. Notably, the activation of anti-apoptotic factors such as B-cell lymphoma-extra large (Bcl-xL), annexin A1, Bak, and myeloid cell leukemia-1 (MCL-1) is the primary cause of delayed neutrophil apoptosis (14, 44). In addition, certain neutrophil subsets (CD16^hi^, CD62L^low^) exhibit suppressive properties by releasing large amounts of the immunosuppressive cytokine IL-10 (45), which is associated with delayed neutrophil apoptosis (46) and also suppresses T-cell proliferation (47). Furthermore, NF-kB-mediated inhibition of caspase-3 and caspase-9, along with impaired phosphorylation and inhibition of caspase-8 catalytic activity, also affects neutrophil apoptosis (48, 49). Overexpression of PD-L1 on septic neutrophils is strongly associated with delayed neutrophil apoptosis, and has been shown to drive lung injury and increase mortality in experimental sepsis, i.e., a cecal ligation and puncture (CLP) model (43). In sepsis, delayed apoptosis allows mature neutrophils to undergo other types of cell death, such as NETosis and autophagy (50, 51). Like NETosis, pyroptosis mediated by caspase-1/11, GSDMD is an essential physiological host defense mechanism. However, excessive neutrophil pyroptosis also contributes to sepsis (52). Overproduction of IL-1β and IL-18 through the classical caspase-1-dependent pathway increases the magnitude of the inflammatory response, suppresses immunity (53), and reduces survival rate (54). Consequently, marked depletion of neutrophils through various cell death pathways accelerates immunosuppression in sepsis.

Monocyte and macrophage apoptosis occurs during the progression of sepsis, potentially leading to immunosuppression and increasing host vulnerability to secondary infections or mortality. Apoptosis in monocytes may reprogram the immune system towards an anti-inflammatory, immunosuppressive response. These monocytes exhibit a reduced capacity to release pro-inflammatory cytokines, such as tumor necrosis factor (TNF), IL-1α, IL-6 and IL-12 against lipopolysaccharide (LPS) and other bacterial inducers, a phenomenon resembling ‘endotoxin tolerance’ that results in poor outcomes (55, 56). Interestingly, the same monocytes are capable of secreting significant levels of anti-inflammatory mediators, such as IL-1 receptor antagonist and IL-10, which correlate with increased rates of nosocomial infection and higher mortality (9). Also, impaired monocytes are linked to decreased antigen-specific lymphocyte proliferation (57). This endotoxin tolerance, along with increased susceptibility to nosocomial infections and elevated mortality, is associated with reduced HLA-DR expression on monocytes and macrophages, referred to as ‘anergy’ (58, 59). Moreover, reports have shown that expression of PD-L1 is increased on the monocytes of septic patients (60) and that this can be used as an independent predictor of mortality (61). In addition, studies have revealed a correlation between reduced monocyte activities and the levels of PD-1 on T lymphocytes (34). Macrophage pyroptosis also contributes to the pathology of septic disseminated intravascular coagulation (DIC) (62), with caspase-11-dependent pyroptosis playing a pivotal role in exacerbating damage and reducing survival (63, 64). Caspase-1-induced monocyte pyroptosis has also been noted in patients with post-traumatic sepsis (65). NETotic-like cell death and macrophage extracellular traps (METs) have also been observed in macrophages (66). Additionally, autophagy influences sepsis progression by affecting senescence, phagocytic capacity, and the activation of inflammatory cytokine release by macrophages (67). Thus, these excessive self-sacrificial processes may facilitate immunosuppression in sepsis.

Dendritic cells (DCs) are dynamic antigen-presenting cells (APCs) that link innate and adaptive immunity and contribute to pathogen recognition, immune response regulation, and inflammation (68, 69). Both conventional dendritic cells (cDCs) and plasmacytoid dendritic cells (pDCs) are highly susceptible to sepsis-induced apoptosis, resulting in significant depletion of DCs in patients with sepsis (70, 71). A study in mouse and other animal models of sepsis has investigated caspase-3-mediated apoptosis of DCs (16). In addition, recent reports have also confirmed the involvement of PD-1 in activating DC apoptosis (72, 73). This intense apoptosis and depletion of DCs not only increases susceptibility to nosocomial infections (74), but also diminishes their functional capabilities (75), resulting in reduced expression of CD40, CD 86, and HLA-DR, and increased section of IL-10 (76, 77). These alterations reflect the tolerogenic state of surviving DCs, which lose their ability to activate effector T cell responses, instead inducing either T cell anergy or Treg cell proliferation (12). Consequently, these immunosuppressive DCs fail to mount an immune response against subsequent bacterial challenges (78). In addition to apoptosis, pDCs can release NET-like extracellular traps (pETs) in response to bacterial infection (79). Furthermore, the loss of autophagy potential in DCs heightens the risk of sepsis (80).

Natural Killer Cells (NK cells) are innate lymphocytes that play a crucial role in coordinating innate and adaptive immune responses in sepsis and defense against pathogen attack (81). NK cells produce IFN-γ during microbial sepsis, and IFN- γ can activate macrophages (71). In sepsis, NK cells undergo extensive apoptosis, significantly decreasing their number in circulation (13). Thus, the low titer of IFN- γ increases the risk of secondary infection. Due to impaired cytokine secretion, the surviving and remaining NK cells cannot correctly induce an immune response against endotoxin. NK cells also lose cytotoxic function, which results in immune suppression (82).

## Sepsis-induced adaptive immune cell death

**Figure 2:**
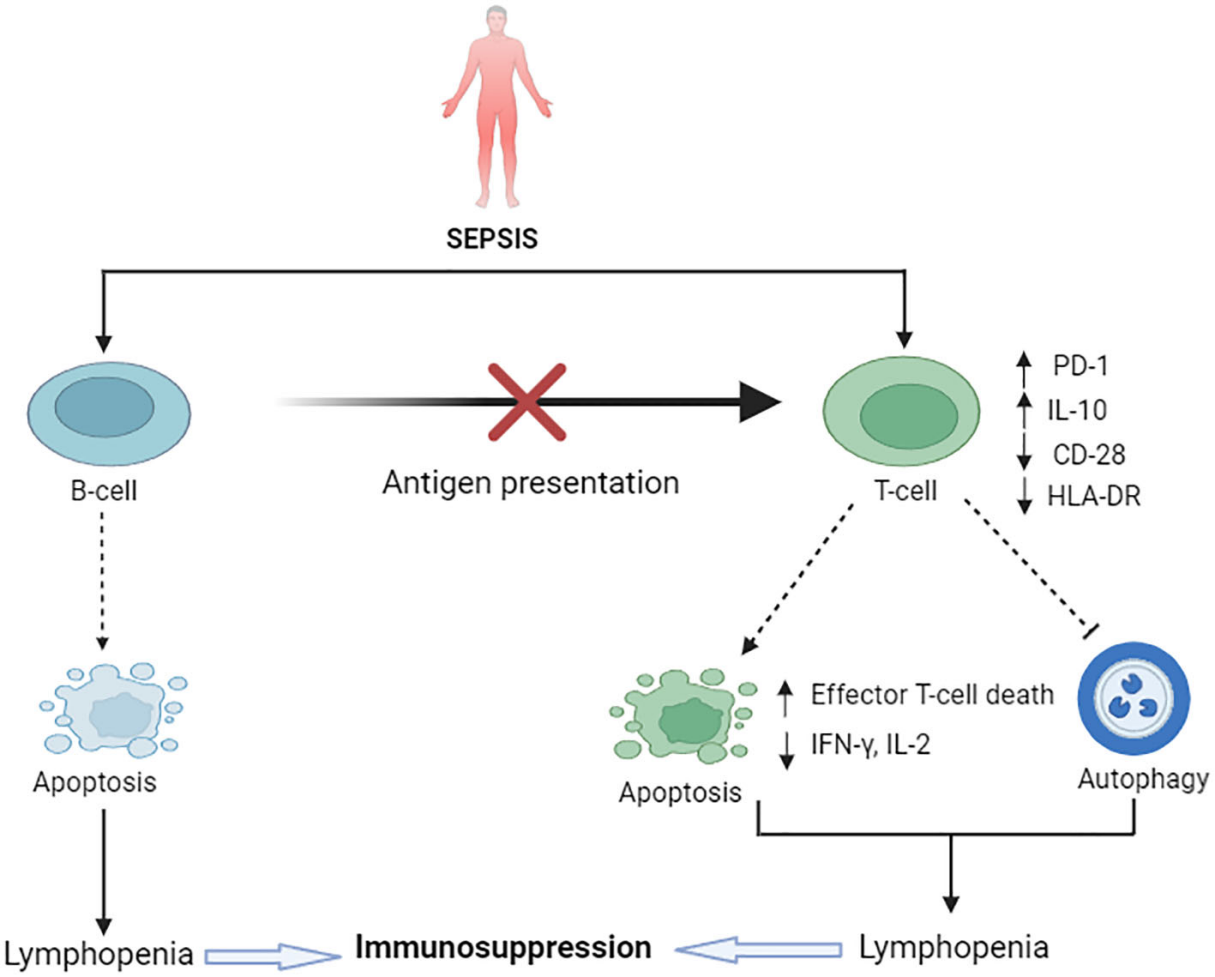
Sepsis-induced immunosuppression: impairment of adaptive immune cell death pathways. Upregulated IL-10 and downregulated CD8 and HLA-DR is notable in sepsis. B-cells undergo apoptosis in sepsis, resulting in a reduction of overall B-cell populations and impairment of the antigen-presenting role of B-cells. Sepsis causes excessive T-cell apoptosis, causing lymphopenia and ultimate immunosuppression. On the contrary, reduced T-cell autophagy in sepsis also contributes to immunosuppression. PD-1 and PD-L-1 play a vital role in this immunosuppression.

The adaptive immune system consists of highly specialized lymphocytes, including T and B lymphocytes. These subpopulations of adaptive immune cells are also susceptible to sepsis-induced cell death. Persistent lymphopenia, a hallmark characteristic in patients with sepsis, is associated with an increased risk of nosocomial infection and a higher risk of mortality (**Figure 2**).

B cells are critical components of both innate and adaptive immunity, with multifunctional roles and diverse phenotypes. Different subpopulations of B cells have specific roles in immunity. For instance, B cells that are activated by pattern recognition receptors (PRRs), referred to as innate response activator (IRA) B cells, augment the antimicrobial response to clear bacteria and induce emergency myelopoiesis by producing GM-CSF and IL-3, respectively (83, 84). Sepsis induces apoptosis in B cells and reduces the diversity of B cell subtypes (85, 86). A growing body of evidence has revealed impaired B cell functions, including diminished antigen presentation to T lymphocytes (87), imperfect interactions with bacterial products (88), and increased secretion of IL-10 (89), which collectively suppress immune responses. Antibody-producing memory B cells, extracellular signal-regulated kinase (ERK)-activation-associated B cells, and CD5^+^ B1 cells are more susceptible to apoptosis compared to other B cell types (85, 86). Sepsis also reduces the number of naïve B cells, triggers B cell exhaustion (90), and impairs the production of IgM (91). While there is no strong evidence directly linking B cell pyroptosis to sepsis, studies using a caspase-1 knockout, IL-1 knockout, and IL-1/IL-18 double knockout mouse models suggest that caspase-1-dependent pyroptosis delays B lymphocyte apoptosis, potentially improving macrophage phenotype and survival rates (92). Further extensive investigation is required to elucidate the relationship between lymphocyte pyroptosis and sepsis.

T cells are the primary actors of the adaptive immune system. Sepsis triggers significant apoptosis of different T cell subsets (93). Specifically, marked apoptosis and reductions in CD4+ and CD8+ T lymphocytes occur in the early phase of sepsis (82, 94). This extensive cell death leads to lymphopenia, which is associated with immunosuppression following the acute resuscitation phase of sepsis. In patients with sepsis, apoptosis of T cells occurs via both intrinsic and extrinsic pathways (95). The interaction of PD-1 with PD-L1 plays a critical pathological role in the immunosuppression observed in sepsis (96). Patients exhibit elevated PD-1 and PD-L1 expression on CD4+ T cells, decreased lymphocyte proliferation, and increased IL-10 secretion (97). In addition, stimulatory molecules such as CD28 and HLA-DR are significantly downregulated in sepsis, reflecting the host’s impaired ability to combat pathogens. Research has shown that the interaction of PD-L1 on APC with PD-1 on T cells disrupts the positive costimulatory signaling of CD28, inhibits T cell proliferation, increases immune effector cell death, reduces cytokine secretion (such as IL-2 and IFN-γ), and ultimately impairs antigen clearance (98). Moreover, a deficiency in T cell autophagy in sepsis contributes to sepsis-induced immunosuppression and increased mortality (99). Another mechanism of immune impairment in the subacute phase of sepsis is T cell anergy (**Figure 1**), which is characterized by the inability of lymphocytes to recognize the cognate antigen, activate, proliferate, and produce cytokines (100). Guinault et al. demonstrated that an expression pattern of the three CD8+ T cell exhaustion markers (2B4, PD-1, and CD160) was strongly associated with the mortality of patients with sepsis (101). To date, there are no reports of lymphocytes releasing NET-like structures.

## Collaboration and interplay among cell death pathways in sepsis

Among the various forms of cell death, apoptosis of immune cells is a central pathophysiological event responsible for sepsis-induced immunosuppression. On one hand, an increased propensity for apoptosis among B cells, T cells, macrophages, and dendritic cells leads to a tolerogenic nature of these cells and a significant reduction in their presence in circulation. On the other hand, delayed apoptosis in neutrophils facilitates alternative forms of cell death, such as autophagy, pyroptosis, and NETosis. Experimental murine models have revealed that sepsis also compromises T cell viability and function by suppressing autophagy and accelerating apoptosis (102). A growing evidence is uncovering the cross-talk among these different cell-death pathways. Notably, there is interaction between apoptosis and autophagy; the cleavage of autophagy-related protein 5 (Atg5) by the protease calpain induces mitochondria-mediated apoptosis through its binding to the anti-apoptotic protein Bcl-xL (103). In septic CD4-Cre/Atg5^f/f^ mice, an increase in the apoptosis of CD+ T cells has been observed, accompanied by upregulation of the pro-apoptotic gene *PDCD1* and downregulation of the anti-apoptotic gene *BCL2* (102). Further research has demonstrated that decreased *ATG5* expression levels are correlated with the severity of sepsis progression and mortality (104), suggesting that the inhibition of autophagy promotes immune cell apoptosis and immunosuppression. Other recent studies highlighted the close association between neutrophil autophagy pathways and increased NET formation in patients with septic-DIC (23, 105). Increased neutrophil autophagy has been noted in survivors of sepsis, and autophagy in healthy neutrophils may stimulate NETs (50). Additionally, autophagy and pyroptosis are inter-connected (106), as are pyroptosis and NETosis, with each having mutual effects. In patients and a mouse model with sepsis, the use of specific inhibitors against primary actors (PAD)2 has been shown to reduce NETosis and macrophage caspase 11-dependent pyroptosis. This inhibition of caspase 11 results in decreased release of inflammatory mediators, increased macrophage counts, enhanced bacterial clearance, and improved survival (107). Targeting GSDMD and PADs could therefore be promising in sepsis therapy, as it couples pyroptosis and NETosis. The ablation of caspase-1/11 in septic mice enhances neutrophil phagocytosis and the levels of inflammatory cytokines, which reflects improved immunity (53).

## Why immunotherapy?

Recent extensive research has broadened our understanding of sepsis pathophysiology, evolving from the traditional view of early inflammation-driven pathology to encompassing concurrent immunosuppression. The discussions above confirm that immunosuppression in sepsis results from the extensive loss of both innate and adaptive immune cells through apoptosis, autophagy, NETosis, pyroptosis, ferroptosis, and necroptosis. Consequently, targeting a single cell death pathway and enhancing host immunity with immunomodulatory agents represents a promising therapeutic strategy to restore impaired host defenses. The following section of this review focuses on immunomodulatory agents aimed at enhancing immune cell function by modulating cell death pathways.

## Current status of clinical therapies

Interleukin-7, primarily derived from stromal cells, is an indispensable hematopoietic cytokine crucial for T cell survival, proliferation, differentiation, and effector functions. It is also an attractive immunoadjuvant therapeutic molecule targeting adaptive immune irregularities in sepsis. Clinical trials have demonstrated that IL-7 is safe and well-tolerated, without inducing toxicities such as cytokine storms or exacerbating inflammation or organ dysfunction. Moreover, IL-7 can inhibit the massive apoptosis of immune-effector cells induced by sepsis and restore the production of IFN-γ, which is essential for the host’s response to invading pathogens (108). A recent clinical trial demonstrated that IL-7 therapy successfully restored depleted CD4+ and CD8+ effector cells by threefold to fourfold in patients with sepsis (109). This pluripotent cytokine can mitigate lymphocyte apoptosis by enhancing the expression of anti-apoptotic proteins such as Bcl-2, CD-28, boosting IFN-γ levels, and increasing TCR diversity, which are typically diminished in patients with sepsis (110, 111). Consequently, recombinant IL-7 therapy not only increases the numbers of CD4+ and CD8+ T cells, but it also reduces Treg cells in circulation, decreasing morbidity and mortality. Interestingly, when IL-7 is administered alongside antiretroviral therapy in patients with HIV exhibiting lymphopenia and immune suppression, it results in reduced PD-1 expression (112). We conclude that IL-7 immunostimulatory therapy, whether used individually or in combination, may represent a promising and potentially protective treatment option for sepsis-induced immunosuppression.

Interleukin-15 promotes the proliferation of memory CD8+ T cells, stimulates dendritic cells, and enhances B cell immunoglobulin production. In a mouse model of sepsis, IL-15 has been shown to attenuate sepsis-induced apoptosis of natural killer (NK) cells, dendritic cells, and CD8+ T cells by increasing the expression of the anti-apoptotic protein Bcl-2, and decreasing the expression of pro-apoptotic proteins Bim and PUMA (113). Additionally, IL-15 enhances IFN-γ production and improves survival in the cecal ligation and puncture (CLP) sepsis model. In cancer trials, the combination of IL-15 and anti-PD-1 therapy has demonstrated reduced IL-10 production and PD-1 expression on CD8+ T cell, augmenting anti-tumor activity (114). Given that IL-15 has shown toxicity in a previous animal study (115), it is important to determine the optimal dosage when employing it as an immunotherapeutic agent in sepsis. Further clinical trials are necessary to evaluate the synergistic potential of IL-15 with other immunotherapeutic agents.

IFN-γ is a key cytokine that is essential for the activation of innate immunity, which is necessary for the clearance of microbial pathogens. However, IFN-γ production is markedly decreased in sepsis. Recombinant IFN-γ therapy in protracted *Staphylococcus aureus* sepsis has been shown to increase monocyte HLA-DR expression and function, as well as enhance bacterial clearance, without any adverse effects (116). Similarly, INF-γ treatment in patients with invasive fungal infections has also been demonstrated to restore HLA-DR expression on leukocytes (117). Although IFN-γ therapy offers potential benefits in patients with sepsis exhibiting immunosuppression by reviving monocyte functions associated with reduced HLA-DR expression, there are no records of it ameliorating T cell defects. Interestingly, the combined application of IFN-γ therapy with the anti-PD-1 antibody, nivolumab, in fungal sepsis has shown promising in restoring immune function and eliminating infection (118). Immunoadjuvant adjunctive IFN-γ therapy, along with IL-7 and anti-PD1/PD-L1, could be beneficial for patients with sepsis, as it has proven impacts on enhancing CD4+ and CD8+ T cell functions.

Fms-like tyrosine kinase-3 ligand (Flt3L), a stem cell growth factor, acts on the class III tyrosine kinase receptor (Flt3R), which is typically expressed on hematopoietic progenitor cells and dendritic cell populations. Enhanced dendritic cell apoptosis and the subsequent impairment of T cell function are common in sepsis pathophysiology. Flt3L treatment has demonstrated effectiveness in promoting the growth and expansion of dendritic cells in both human (119) and mouse models (120). In addition, Flt3L therapy in models of burn injury and sepsis not only increases dendritic cell populations but also enhances neutrophil antimicrobial functions and improves survival (121). A recent study using a mouse model of burn injury and sepsis has shown that Flt3L treatment mitigates T cell depletion, restores CD28 expression on CD4+ and CD8+ T cells, and increases IFN-γ production by CD8+ T cells, thereby reducing organ injury markers and enhancing survival (122). Flt3L also suppresses PD-L1 expression on APCs, such as dendritic cells, macrophages and monocytes. A research group has revealed that Flt3 can mitigate oxidative stress and protect cardiomyocytes from apoptotic death through the regulation of Bcl-2 family proteins (123). Thus, Flt3 is hypothesized to reduce T cell apoptosis in sepsis. Further investigation is warranted to explore the synergistic potential of Flt3 therapy with other established therapeutics, such as IL-7, in the management of sepsis.

Granulocyte macrophage colony stimulating factor (GM-CSF), a hematopoietic growth factor, enhances the production of neutrophils and monocytes, enhances monocyte survival, and restores TNF production, thereby helping to prevent nosocomial infections and mitigate immunosuppression (124). GM-CSF therapy increases the expression of HLA-DR on monocytes, facilitates bacterial clearance, and contributes to more ventilation-free days and reduced stays in the intensive care unit (125). However, a meta-analysis by Bo et al. found no evidence supporting the routine use of G-CSF or GM-CSF in patients with sepsis (126).

PD-1 receptor system acts as a negative regulator of the immune response. In sepsis, there is an overexpression of inhibitory receptors PD-1 on B and T lymphocytes and PD-L1 and PD-L2 on epithelial cells, endothelial cells, and APCs. This overexpression leads to decreased cytokine secretion, increased apoptotic cell death, immunosuppression, and, eventually, deleterious outcomes. A high serum soluble form of PD-L1 (sPD-Ll) has also been detected in patients with sepsis, and is correlated with disease severity and poor clinical outcomes (127). The PD-1/PD-L1 axis, targeted by immune check point inhibitor antibodies, is gaining attention as an immunotherapeutic approach in sepsis due to its successful application in the treatment of infectious diseases and regression of advanced-stage cancers (128, 129). In addition, anti-PD-1/PD-L1 therapy has also been shown to increase the expression of CD28 on proliferating peripheral CD8+ T cells following treatment. Mice models and *ex vivo* clinical studies of patients with sepsis have shown that blockade of PD or PD-L1 plays a significant role in reversing immune defects caused by sepsis (130). Furthermore, anti-PD-L1 treatment has been shown to promote apoptosis in septic neutrophils in mice models (131). The immunosuppressive properties of septic neutrophils, monocytes, and macrophages can also be reversed by blocking either PD-1 or PD-L1 (34, 132). Additionally, treatment with anti-PD1 antibodies enhances DC survival in sepsis (72). An anti-PD-1 antibody nivolumab, i.e., immune checkpoint inhibitor has been evaluated the safety, tolerability, pharmacokinetics, and pharmacodynamics at the phase 1b (133) and the phase 1/2 study (134). Taken together, these findings suggest that targeting the PD-1/PD-L1 axis with immunoadjuvant therapy represents a promising approach to reverse sepsis-induced immunosuppression.

Autophagy has recently gained attention in the field of critical care due to its role in regulating cell apoptosis. Enhancing T cell autophagy may alleviate sepsis-induced immunosuppression by modulating apoptosis (102). Additionally, inhibiting NETosis represents another potential strategy for sepsis management. A recent study in rodent models of sepsis has shown that NET inhibition using Cl-amidine, a PAD4 inhibitor, is effective (135). Another study has demonstrated the inhibition of PAD4 and NETosis in both mice and humans using YW3-56 as an inhibitor (136). Moreover, disulfiram, an FDA-approved drug, targets GSDMD activation, blocking pyroptosis and NETosis, thereby improving survival in mice (137). **Table 1** summarizes some preclinical treatments against immunosuppression in sepsis.

**Table 1:** Preclinical treatments for immunosuppression during sepsis.

Immune Modulator	Outcome	Reference
IL-7	Inhibit sepsis-induced massive immune cell apoptosis Enhance production of CD4+ T and CD8 T cells Increase T-cells infiltration to sites of infection Boost IFN-γ production	(108–111)
IL-15	Prevent sepsis-induced apoptosis of CD8 T cells, NK cells, and DCs	(113)
IFN-γ	Upregulate monocyte expression of HLA-DR, increase numbers of IL-17 producing CD4+ T cells	(116, 117)
Flt3L	Promote DCs Promote neutrophil antimicrobial function Suppresses PD-L1 expression on APCs Augment IFN-γ production	(119, 121, 122)
GM-CSF	Increase production of neutrophils and monocytes or macrophages and reduce cell death Augment the expression of HLA-DR on monocytes	(124, 125)
PD-1/PD-L1	Protect immunosuppressive properties of septic neutrophils, monocytes, and macrophages Anti-apoptotic effects to prevent loss of protective function of NK cells Prevent lymphocyte apoptosis and reverse monocyte dysfunction Initiate neutrophil apoptosis Restore monocyte HLA-DR antigen expression and lymphocyte count	(34, 131–134, 138)

## Adverse reactions of immunotherapy

The main concern of immune therapy in sepsis is the risk of hyper-inflammatory response that can increase the severity of the disease, even death. An animal study reported that IL-15 immunotherapy has a toxic effect, causing liver injury and cachexia (115, 139). However, clinical trials of IL-7 and IFN-γ therapy showed no adverse reactions like cytokine storms or exacerbating inflammation and were well tolerated (108, 116). Although PD-1/PD-L1 is a promising therapy, PD-1 deficiency is also related to the occurrence of autoimmune diseases such as lupus-like syndromes, *de novo* type 1 diabetes, and dilated cardiomyopathy (140, 141). That is why the timing and duration of PD-1/PD-L1 blocking should be done with proper attention. In conclusion, patients’ immune status, optimal dosage, timing, and personalized approach should be considered before starting the clinical application of immunotherapy to avoid adverse reactions.

## Conclusions and future directions

Immune cells employ mechanisms such as autophagy in B cells and T cells, as well as NETosis and pyroptosis in neutrophils and macrophages, initially to protect the host. However, when overactivated, these protective effects can become detrimental. Dysregulated immune cell death, including apoptosis, autophagy, NETosis, and pyroptosis, along with impaired immune status, contributes significantly to the pathophysiology of sepsis. To effectively address sepsis, it is imperative to explore other types of cell death and their underlying mechanisms. In addition, elucidating the cross-talk among apoptosis, autophagy, pyroptosis, and NETosis is necessary. These cell death processes augment inflammation, deplete immune cells, and lead to immunosuppression. Targeting, closely monitoring, and regulating these cell death mechanisms could offer a promising approach to treating patients with sepsis, ultimately improving survival. In this review, we have highlighted numerous immunoadjuvant therapeutic agents that possess significant potential to enhance suppressed immunity in sepsis. While immunotherapy represents a promising strategy against sepsis, the broad variations in immune status among patients must be carefully considered for clinical applications. Biomarker-guided stratification and a personalized approach for each patient are imperative. Furthermore, combination therapies may offer a higher success rate in countering immunosuppressive sepsis in the future.
